# Instrumental variable estimation for a time-varying treatment and a time-to-event outcome via structural nested cumulative failure time models

**DOI:** 10.1186/s12874-021-01449-w

**Published:** 2021-11-25

**Authors:** Joy Shi, Sonja A. Swanson, Peter Kraft, Bernard Rosner, Immaculata De Vivo, Miguel A. Hernán

**Affiliations:** 1 Department of Epidemiology, Harvard T.H. Chan School of Public Health, Boston, MA USA; 2 The CAUSALab, Harvard T.H. Chan School of Public Health, Boston, MA USA; 3 Department of Epidemiology, Erasmus Medical Center, Rotterdam, The Netherlands; 4 Department of Biostatistics, Harvard T.H. Chan School of Public Health, Boston, MA USA; 5 Channing Division of Network Medicine, Department of Medicine, Brigham and Women’s Hospital and Harvard Medical School, Boston, MA USA

**Keywords:** Mendelian randomization, Instrumental variable, Structural nested models, G-estimation

## Abstract

**Background:**

In many applications of instrumental variable (IV) methods, the treatments of interest are intrinsically time-varying and outcomes of interest are failure time outcomes. A common example is Mendelian randomization (MR), which uses genetic variants as proposed IVs. In this article, we present a novel application of g-estimation of structural nested cumulative failure models (SNCFTMs), which can accommodate multiple measures of a time-varying treatment when modelling a failure time outcome in an IV analysis.

**Methods:**

A SNCFTM models the ratio of two conditional mean counterfactual outcomes at time *k* under two treatment strategies which differ only at an earlier time *m*. These models can be extended to accommodate inverse probability of censoring weights, and can be applied to case-control data. We also describe how the g-estimates of the SNCFTM parameters can be used to calculate marginal cumulative risks under nondynamic treatment strategies. We examine the performance of this method using simulated data, and present an application of these models by conducting an MR study of alcohol intake and endometrial cancer using longitudinal observational data from the Nurses’ Health Study.

**Results:**

Our simulations found that estimates from SNCFTMs which used an IV approach were similar to those obtained from SNCFTMs which adjusted for confounders, and similar to those obtained from the g-formula approach when the outcome was rare. In our data application, the cumulative risk of endometrial cancer from age 45 to age 72 under the “never drink” strategy (4.0%) was similar to that under the “always ½ drink per day” strategy (4.3%).

**Conclusions:**

SNCFTMs can be used to conduct MR and other IV analyses with time-varying treatments and failure time outcomes.

**Supplementary Information:**

The online version contains supplementary material available at 10.1186/s12874-021-01449-w.

## Introduction

Instrumental variables (IVs) provide an approach to consistently estimate an average causal effect of a treatment on an outcome, even in the presence of unmeasured confounding. In many randomized trial designs, IV methods allow one to estimate the per-protocol effect by using randomization assignment as an IV [1]. In observational studies, an increasingly popular application of IVs is Mendelian randomization (MR), in which genetic variants are used as proposed IVs [2, 3]. In many IV studies, including those which use MR, the treatments of interest (e.g. blood lipids, smoking, alcohol intake) are intrinsically time-varying and many outcomes of interest are failure time outcomes. However, conventional IV methods were designed to handle time-fixed treatments and IV methods for failure time outcomes are less commonly used in practice [4]. Thus, there is a mismatch between the goal of these studies and the availability of IV methods.

We have previously described extensions of IV methods based on g-estimation of structural mean models to incorporate time-varying treatments in MR analyses [5]. Others have described structural nested accelerated failure time [6] and structural nested cumulative survival [7–9] models to incorporate failure time outcomes in an IV analysis with time-varying treatments. However, unlike our approach, parameter estimation in structural nested accelerated failure time models requires artificial censoring (which is statistically inefficient and makes estimation numerically difficult because estimating equations are not differentiable), and structural nested cumulative survival models only compare static treatment [9].

Structural nested cumulative failure time models (SNCFTMs) overcome both of these limitations [10]. SNCFTMs have been previously discussed as a method of estimating the causal effect of a time-varying treatment on a failure time outcome under the sequential exchangeability assumption that all time-varying confounders have been measured and that failure is rare under all possible treatment values [10]. Here we describe an adaptation of this use of SNCFTMs that, under the same rare failure assumption, replaces the sequential exchangeability assumption with IV-type assumptions. We first introduce notation and describe SNCFTMs when estimating the parameters using an IV. Next, we examine the performance of the proposed method in simulation studies. Then, we present an MR study estimating the effect of alcohol intake on endometrial cancer risk based on data from the Nurses’ Health Study I.

## Methods

### Notation and identifying assumptions

Let *k* = 0, 1, 2, …, *K* + 1 denote a time interval where *k* = 0 denotes start of follow-up. For each individual, let *Z* represent the value of a time-fixed instrument (e.g., germline genetic variants in MR studies), *A*_*k*_ the treatment value during interval *k*, and *Y*_*k*_ an indicator (1: yes, 0: no) for the outcome before start of interval *k* = 1, 2, …, *K* + 1. We use an overbar to represent history from time 0, that is ${\overline{A}}_k=\left({A}_0,{A}_1,\dots, {A}_k\right)$, and an underbar to represent treatment up to the end of the study, that is ${\underline A}_k=\left(A_k,A_{k+1},\dots,A_K\right)$.

A static treatment strategy *g* is defined as $g\equiv {\overline{g}}_K\equiv \left({a}_0,{a}_1,\dots, {a}_K\right)$ where treatment *a*_*k*_ is assigned to each individual at time *k*. For example, for a dichotomous treatment, the strategy “never treat” is represented by $g=\overline{0}$ and the strategy “always treat” is represented by $g=\overline{1}$. In failure time settings, the strategy “always treat” is more precisely specified as “always treat before failure” and thus can be viewed as a dynamic strategy $g\equiv {\overline{g}}_K\equiv \left({g}_0,{g}_1,\dots, {g}_K\right)$, where *g*_*k*_ = 1 when *Y*_*k* − 1_ = 0 and *g*_*k*_ = 0 otherwise. In this paper, we will also consider the strategy “receive the treatment actually received through *k* but no treatment thereafter”, which is represented by $g=\left({\overline A}_k,\underline0\right)$*.*

Let ${Y}_k^g$ represent the counterfactual outcome at time *k* had they followed the treatment strategy *g*. By consistency, the counterfactual outcome ${Y}_k^g$ is equal to the observed outcome *Y*_*k*_ among individuals whose observed treatment history is equal to that specified by *g* between times 0 and *k* − 1.

The instrumental variable *Z* is defined by meeting the three instrumental conditions: (1) the instrument and the treatment are associated, or $Z\;\coprod\;A_k$ does not hold for any *k* (a stronger version is often needed for estimation, e.g., for linear models, the *Z* and *A*_*k*_ need to be correlated); (2) the instrument affects the outcome only through the treatment, or ${Y}_{i,k}^{z,g}={Y}_{i,k}^{\mathrm{z}\prime, g}={Y}_{i,k}^g$ for all individuals *i*, *k*, *z*, *z*′, *g*; and (3) there are no shared causes, or other sources of lack of exchangeability, between the instrument and the outcome, or $Z\;\coprod Y_k^{z,g}$ for all *z*, *k*, *g*. The last two conditions, taken together, imply exchangeability between the instrument and the counterfactual outcome under a given treatment strategy, $Z\;\coprod\;Y_k^g$.

The three instrumental conditions alone are generally insufficient to obtain a point estimate. To do so, we can make a fourth assumption of homogeneity. One version of this assumption asserts that the instrument *Z* does not modify the effect of the treatment *A*_*k*_ on the outcome *Y*_*k* + 1_ on the multiplicative scale. As we describe below, this assumption precludes us from including a product term between the instrument and the treatment in the model.

### G-estimation of structural nested cumulative failure time models with an instrumental variable

Let $\mathrm E\left[Y_k^g\right]$ represent the counterfactual risk of developing the outcome by time *k* had everyone followed the treatment strategy *g.* SNCFTMs compare the counterfactual risks at *k* under the strategies $\left({\overline A}_m,\underline0\right)$ and $\left({\overline A}_{m-1},\underline0\right)$, for each time *m* < *k*, among individuals who are free of the outcome through *m* (i.e., *Y*_*m*_ = 0) and had treatment history ${\overline{A}}_m$ and the same covariate history through *m*. When adjusting for confounders, “covariate history” means confounder history [10]; when using instrumental variable estimation, “covariate history” means the instrument *Z*. “Covariate history” may additionally include instrument-outcome confounders or effect modifiers to allow for a weaker variation of the instrumental conditions or the homogeneity assumption—a point we will describe further in the discussion.

Specifically, an SNCFTM models the ratio of two counterfactual cumulative risks at time *k* under treatment strategies that differ only at time *m* for each time *m* < *k*:

$$\exp\left[\gamma_k\left({\overline A}_m,Z;\psi\right)\right]=\left\{\begin{array}{lc}\frac{\mathrm E\left[Y_k^{\left({\overline A}_m,\underline0\right)}\vert{\overline A}_m,Z,Y_m=0\right]}{\mathrm E\left[Y_k^{\left({\overline A}_{m-1},\underline0\right)}\vert{\overline A}_m,Z,Y_m=0\right]}&\mathrm{if}\;Y_m=0\\1&\mathrm{if}\;Y_m=1\end{array}\right.$$

where ${\gamma}_k\left({\overline{A}}_m,Z;\psi \right)$ is a function of the treatment history through *m* and the instrument, indexed by the parameter *ψ*. That is, the SNCFTM models the conditional effect of a “blip” of treatment at time *m* on outcome at time *k*. Hence, ${\gamma}_k\left({\overline{A}}_m,Z;\psi \right)$ is also referred to as a “blip function”. This model is semi-parametric as it allows for the counterfactual cumulative risks to remain unspecified; however, under a non-saturated SNCFTM, the choice of the blip function will impose restrictions on the assumed distribution of the data. For example, the simple blip function

$$\gamma_k\left({\overline A}_m,Z;\psi\right)=\psi A_m$$

assumes the effect of treatment at time *m* on outcome at time *k* to be constant for all *m* < *k* and across levels of the instrument *Z*. Note that SNCFTMs with an instrument are necessarily non-saturated because the fourth condition of homogeneity (i.e., the effect of *A*_*m*_ on *Y*_*k*_ is constant across levels of *Z* among both the treated and the untreated) implies the absence of product terms between the instrument *Z* and the treatment *A*_*m*_ [11]. Therefore, for our IV analysis, the blip function cannot depend on the instrument Z, or ${\gamma}_k\left({\overline{A}}_m,Z;\psi \right)={\gamma}_k\left({\overline{A}}_m;\psi \right)$. The blip function should be chosen such that $\exp \left[{\gamma}_k\left({\overline{A}}_m;0\right)\right]=1$ when treatment at time *m* has no effect on outcome at time *k* [10]. For our analyses, we use

$$\exp \left[{\gamma}_k\left({\overline{A}}_m;\psi \right)\right]=1+\frac{\exp \left(\psi {A}_m\right)-1}{k-m}$$

in which *ψ* = 0 corresponds to no effect, *ψ* < 0 to protective effect, *ψ* > 0 to harmful effect, and allows for the effect of *A*_*m*_ to diminish as time since *m* increases. The choice of blip function should be based on a priori subject matter knowledge, although one could also consider fitting models under a suite of possible blip functions as a sensitivity analysis to assess how that affects one’s conclusions. Other choices of blip functions have been previously described [10].

The interpretation of the parameter *ψ* depends on the choice of blip function [10]. Under certain conditions, as described in the next section, the parameter *ψ* can be mapped into the counterfactual risks under treatment strategies of interest, which have a natural interpretation for causal inference. In the remainder of this section, we describe g-estimation of *ψ*. In the next section, we describe how to use the estimate to compute the risks under the strategies of interest.

G-estimation has been previously described under the assumption of no unmeasured treatment-outcome confounders [10]. For IV estimation, the estimating function is [10, 11]:

$$U\left(\psi^\dagger;Z\right)=\sum\nolimits_{m=0}^K\left(1-Y_m\right)\sum\nolimits_{k=m+1}^{K+1}\left(Z-\mathrm E\left[Z\vert Y_m=0\right]\right)H_{m,k}\left(\psi^\dagger\right)$$

where *H*_*m*, *k*_(*ψ*^†^) is defined as

$$H_{m,k}\left(\psi^\dagger\right)=\left\{\begin{array}{lc}Y_k\exp\left(-\sum\nolimits_{j=m}^{k-1}\gamma_j\left({\overline A}_j;\psi^\dagger\right)\right)&\mathrm{if}\;Y_m=0\\1&\mathrm{if}\;Y_m=1\end{array}\right.$$

We use *ψ*^†^ to denote candidate values of the true value *ψ* where $\mathrm E\left[H_{m,k}\left(\psi^\dagger\right)\vert{\overline A}_m,Z\right]$ is equal to the mean counterfactual outcome, $\mathrm E\left[Y_k^{\left({\overline A}_{m-1},\underline0\right)}\vert{\overline A}_m,Z\right]$, when *ψ*^†^ is equal to *ψ*.

Under the IV assumptions, the value *ψ*^†^ that solves E[*U*(*ψ*^†^; *Z*)] = 0 is our g-estimate [11]. The equation can be numerically solved, as previously described, using the Newton-Raphson procedure [10]. The 95% confidence interval for $\hat{\psi}$ can be obtained by bootstrapping. Selection bias due to censoring during follow-up can be addressed by inverse probability weighting, as has been previously described [10].

### Computing marginal risks under two treatment strategies

The parameter *ψ* of the SNCFTM can be used to calculate the counterfactual risks under a given treatment strategy [10]. The risk $\mathrm {E}\left[{Y}_k^{\overline{0}}\right]$ under the “never treat” strategy $\overline{0}$ is obtained by “removing” the effect of an individual’s non-zero treatment at each time period *k* from the end of the study period (*k* = *K* + 1) to the beginning (*k* = 0). This calculation, referred to as “blipping down” procedure, is carried out using the formula that is a function of the observed data when ${\gamma}_k\left({\overline{A}}_m;\psi \right)$ is known

$$\mathrm {E}\left[{Y}_k^{\overline{0}}\right]=\mathrm {E}\left[{Y}_k\prod\nolimits_{m=0}^{k-1}\exp\left[-\gamma_k\left({\overline {A}}_m;\psi\right)\right]\right]$$

The risk $\mathrm{E}\left[{Y}_k^g\right]$ under the “always treat” treatment strategy *g* is obtained by “adding” the effect of treatment to $\mathrm {E}\left[{Y}_k^{\overline{0}}\right]$ from the beginning of the study period (*k* = 0) to the end of the study period (*K* + 1). Assuming no effect measure modification of the treatment by time-varying covariates on the multiplicative scale, we can estimate this quantity using only $\mathrm {E}\left[{Y}_k^{\overline{0}}\right]$ and ${\gamma}_k\left({\overline{A}}_m;\psi \right)$. Under this assumption, this calculation, referred to as “blipping up”, is carried out using the formula

$$\mathrm {E}\left[{Y}_k^g\right]=\sum\nolimits_{j=0}^{k-1}\mathrm {E}\left[Y_{k-j}^{\overline{0}}\right]\times t_{\overline {a}}\left(k,j,-1\right)$$

where ${t}_{\overline{a}}\left(k,j,i\right)$ for *i* =  − 1, 0, 1, …*k* − 2 are recursively defined as:

$${t}_{\overline{a}}\left(k,0,k-2\right)={e}_{\overline{a}}\left(k,k-1\right)$$

$${t}_{\overline{a}}\left(k,j,k-1-\left[s+1\right]\right)={t}_{\overline{a}}\left(k,j,k-1-s\right)\times {e}_{\overline{a}}\left(k-j,k-1-s\right),\mathrm{for}\ \mathrm{j}<\mathrm{s}$$

$${t}_{\overline{a}}\left(k,s,k-1-\left[s+1\right]\right)=\left[1-{\sum}_{j=0}^{s-1}{t}_{\overline{a}}\left(k,j,k-1-s\right)\right]\times {e}_{\overline{a}}\left(k-s,k-1-s\right)$$

with *j* ≤ *s* and ${e}_{\overline{a}}\left(p,m\right)=\exp \left[{\gamma}_p\left({\overline{a}}_m;\psi \right)\right]$. These recursive definitions of ${t}_{\overline{a}}\left(k,j,i\right)$ weight the probability of developing the outcome at each time *j* < *k* by the cumulative probability of survival through *j* − 1. In the presence of effect measurement modification by time-varying covariates, estimation of nuisance functions, in addition to $\mathrm{E}\left[{Y}_k^{\overline{0}}\right]$ and ${\gamma}_k\left({\overline{A}}_m;\psi \right)$, are required for the calculation of $\mathrm{E}\left[{Y}_k^g\right]$[10].

### Analysis of case-control data using SNCFTMs

In some cases, data on treatment and outcome are available for all individuals in the study cohort, but the proposed instrument, such as a given genetic variant, is difficult or expensive to measure in the full cohort. One solution is to limit the measurement of the instrument to cases (individuals who develop the outcome during the follow-up) and incidence-density sampled controls.

Case-control sampling allows us to consistently estimate the parameter *ψ* of the SNCFTM and the marginal risks under static treatment strategies. To see why, note that only the cases contribute to the sum in the estimating equation because *H*_*m*, *k*_(*ψ*^†^) is equal to 0 for all *m* and *k* among individuals who remain free of the outcome over follow-up. Therefore, if all cases or a random sample of the cases are included in the case-control sample, then the g-estimate $\hat{\psi}$ remains unbiased as long as E[*Z*| *Y*_*m*_ = 0] is correctly estimated in the full cohort or in randomly selected controls that are representative of the underlying at-risk population that gave rise to the cases. As such, the case-control sample is sufficient to estimate *ψ*, even when sampling fractions are unknown.

Once the g-estimate $\hat{\psi}$ is obtained, the marginal counterfactual risks under static treatment strategies can be estimates as described in the previous section. This is the case because the blip function cannot be a function of the instrument under the homogeneity assumption, and the data on treatment and outcome is available for the full cohort.

## Simulation study

**Fig. 1:**
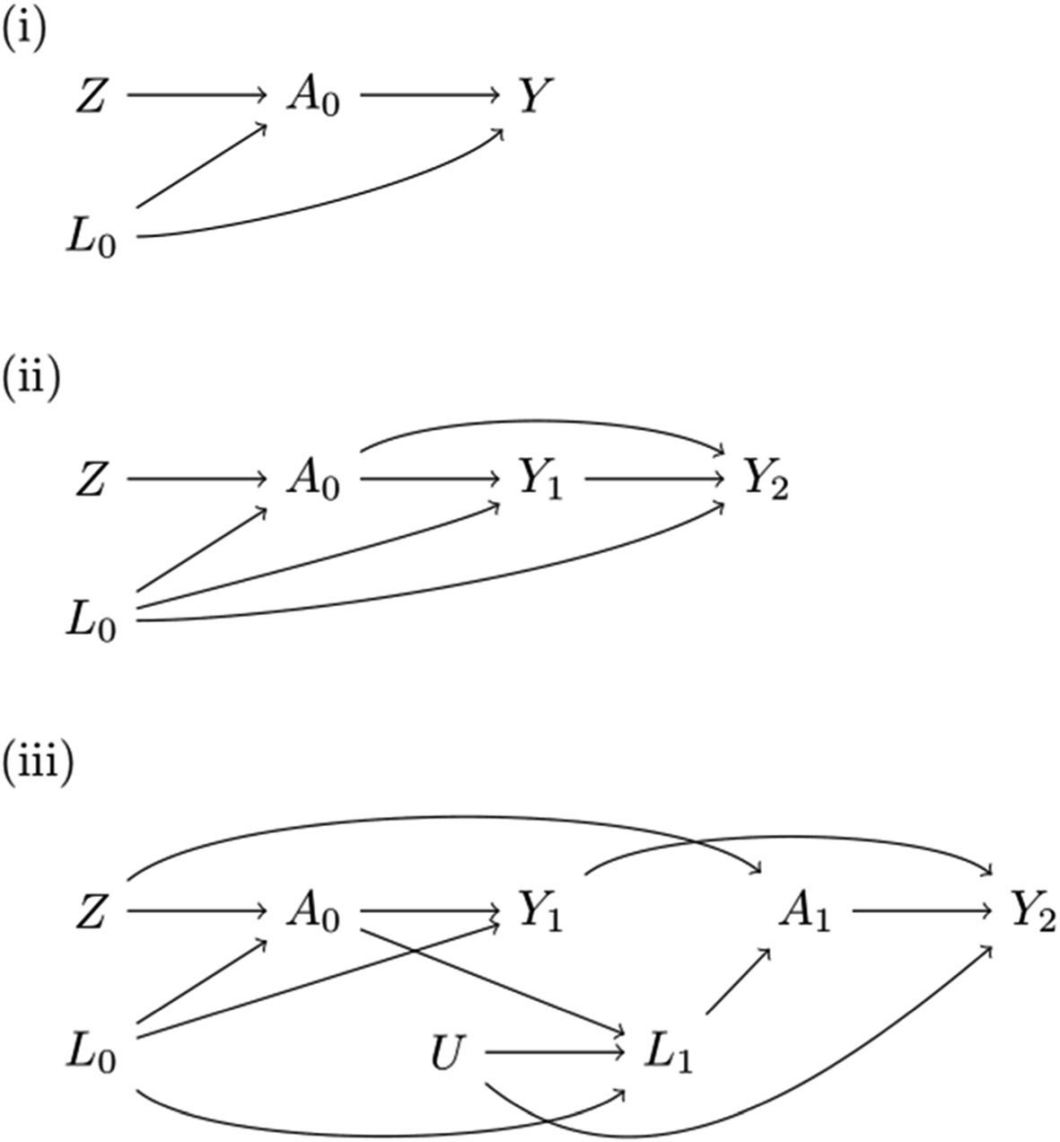
Causal diagrams depicting the relationship between a time-fixed instrument and (**i**) a time-fixed exposure and outcome; **ii** a time-fixed exposure and a time-varying outcome; **iii** a time-varying exposure and outcome

We simulated datasets of 25,000 individuals compatible with the three scenarios shown in Fig. 1: (i) a time-fixed treatment *A*_0_, a time-fixed outcome *Y*_1_ and a time-fixed confounder *L*_0_, (ii) a time-fixed treatment *A*_0_, a time-varying outcome *Y*_1_, *Y*_2_ (where *Y*_*k*_ is an indicator for having developed the event by time *k*) and a time-fixed confounder *L*_0_, and (iii) a time-varying treatment *A*_0_, *A*_1_, a time-varying outcome *Y*_1_, *Y*_2_ and a time-varying confounder *L*_0_, *L*_1_. In all settings, there was a causal instrument *Z*. For simplicity, we assume no loss to follow-up and considered all variables as binary. We used the following data-generating model:

$$\Pr \left({L}_0=1\right)=0.25$$

$$\Pr \left({L}_1=1|{L}_0,{A}_1\right)=0.25+0.25{L}_0+0.25{A}_1$$

$$\Pr \left(Z=1\right)=0.5$$

$$\Pr \left({A}_k=1|Z,{L}_k\right)={\alpha}_{ZA}Z+0.5{L}_k$$

$\Pr \left({Y}_{k+1}=1|{A}_k,{L}_k,{Y}_k=0\right)=\mathrm{expit}\left(\log \left(\frac{\lambda }{1-\lambda}\right)+{\alpha}_{AY}{A}_k+0.5{L}_k\right)$ for scenarios (i) and (iii).

$\Pr \left({Y}_{k+1}=1|{A}_0,{L}_0,{Y}_k=0\right)=\mathrm{expit}\left(\log \left(\frac{\lambda }{1-\lambda}\right)+{\alpha}_{AY}{A}_0+0.5{L}_0\right)$ for scenario (ii).

where *α*_*ZA*_ = 0.25, *α*_*AY*_ = 0 when data was generated under the null and *α*_*AY*_ = 0.5 otherwise; and baseline constant hazards (*λ*) took on values of 5, 10 and 25%. Additional simulations were conducted with datasets of 10,000 or 50,000 individuals, and with varying strengths of association between the instrument and the exposure (*α*_*ZA*_ = 0.10 and *α*_*ZA*_ = 0.45) (Supplementary Fig. 1). Also, to create a case-control study, we selected all individuals who developed the outcome as cases and randomly sampled two controls per case.

We fit a SNCFTM defined by the blip function $\exp \left[{\gamma}_k\left({\overline{A}}_m;\psi \right)\right]=1+\frac{\exp \left(\psi {A}_m\right)-1}{k-m}$ and g-estimated the parameters of the model using three approaches:

1. Adjusting for confounder *L*
2. Using the instrumental variable *Z*
3. Neither adjusting for confounder *L* nor using the instrumental variable *Z*

We calculated differences and ratios in marginal risks under the “never treat” strategy and the “always treat” strategy by using the $\hat{\psi}$ estimates from each SNCFTM and by applying the g-formula, a generalization of standardization to time-varying treatments and confounders. When the data were simulated under the null, 0 was the true *ψ* parameter. When the data were simulated not under the null, we considered the mean $\hat{\psi}$ value obtained by adjusting for confounder *L* as the true value of the parameter *ψ*.

**Fig. 2:**
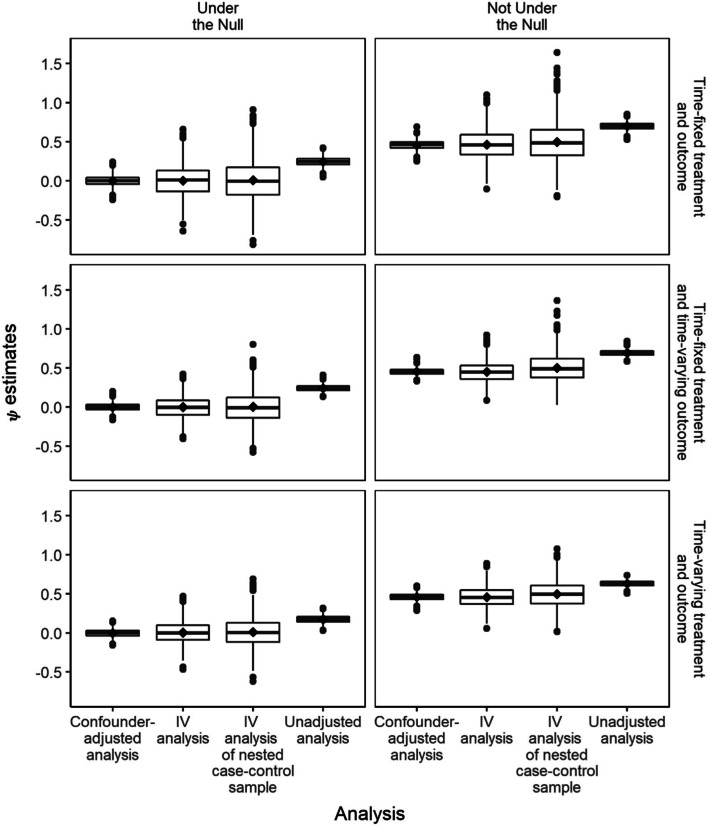
Distributions of psi estimates across 1000 iterations using different g-estimation approaches under different data-generating mechanisms with *λ* = 5%. The lower and upper hinges correspond to the 25th and 75th percentile. The lower and upper whiskers extend from the hinge to the smallest and largest values no further than 1.5*IQR from the hinge, where IQR is the interquartile range. The median is represented by the line between the hinges, and the mean is represented by the diamond point symbol

Distributions of $\hat{\psi}$ estimates across simulated iterations with *λ* = 5% are given in Fig. 2 and Table 1. Compared with the mean $\hat{\psi}$ estimates from SNCFTMs which adjusted for confounder *L*, the mean $\hat{\psi}$ estimates from SNCFTMs which used an IV approach were similar. The variance of the IV estimates was larger than that of the confounding-adjusted estimates. There was additional loss in efficiency when IV was applied to data from a case-control design compared to a full cohort due to increased variability in estimating E[*Z*| *Y*_*m*_ = 0]. As expected, $\hat{\psi}$ estimates were very biased when we neither appropriately adjusted for confounding nor used an IV, with the bias ranging between 0.23 to 0.27. Estimates for marginal risk differences and risk ratios were similar between the SNCFTM approach and the g-formula approach (Supplementary Fig. 2A-B). As the baseline hazard increased, the SNCFTM approach resulted in an overestimate of the risk differences and the risk ratios compared to using the g-formula (Supplementary Fig. 3A-B).

**Table 1:** Mean, standard deviation (SD), bias and mean squared error (MSE) of psi estimates using different g-estimation approaches under different data-generating mechanisms

DAG	Under the null	Measure	Confounding-adjusted analysis	IV analysis	IV analysis of nested case-control sample	Unadjusted analysis
Time-fixed exposure and outcome	Yes	Mean	0.0013	0.0044	0.0102	0.2468
		SD	0.0655	0.2001	0.2665	0.0563
		Bias	+0.0013	+0.0044	+ 0.0102	+ 0.2468
		MSE	0.0043	0.0400	0.0711	0.0641
	No	Mean	0.4591	0.4655	0.5010	0.7016
		SD	0.0579	0.1885	0.2563	0.0478
		Bias	0.0000	+0.0064	+0.0419	+0.2424
		MSE	0.0034	0.0355	0.0674	0.0611
Time-fixed exposure and time-varying outcome	Yes	Mean	−0.0024	−0.0018	0.0021	0.2410
		SD	0.0473	0.1446	0.1950	0.0401
		Bias	−0.0024	− 0.0018	+0.0021	+0.2410
		MSE	0.0022	0.0209	0.0380	0.0597
	No	Mean	0.4523	0.4489	0.5015	0.6923
		SD	0.0417	0.1365	0.1833	0.0348
		Bias	0.0000	−0.0033	+0.0492	+0.2400
		MSE	0.0017	0.0186	0.0360	0.0588
Time-varying exposure and outcome	Yes	Mean	−0.0018	0.0061	0.0122	0.1717
		SD	0.0539	0.1428	0.1861	0.0470
		Bias	−0.0018	+0.0061	+0.0122	+0.1717
		MSE	0.0029	0.0204	0.0348	0.0317
	No	Mean	0.4600	0.4588	0.4974	0.6316
		SD	0.0448	0.1293	0.1706	0.0378
		Bias	0.0000	−0.0012	+0.0374	+0.1716
		MSE	0.0020	0.0167	0.0305	0.0309

### Application: the effect of alcohol intake on endometrial cancer

Alcohol intake may increase endometrial cancer risk by increasing estrogen levels or may decrease endometrial cancer risk by improving insulin sensitivity and reducing fasting insulin concentrations [12, 13]. To estimate this effect, we emulated a target trial of alcohol intake interventions among middle-age women using observational data from the Nurses’ Health Study (NHS), a prospective study of female registered nurses [14]. Below we summarize the protocol of the target trial and describe how to emulate each of its components using the NHS observational data.

### Target trial specification

The eligibility criteria for the women in the target trial would be 45–48 years of age, no history of cancer (except for non-melanoma skin cancer), no history of alcoholism, and an intact uterus. The two (static) strategies to be compared would be (1) “never drink”, or (2) “always ½ drink per day, unless an absolute contraindication for moderate alcohol consumption arises”. We considered a standard drink to contain 14 g of ethanol [15]. Eligible women would be randomly assigned to one of the strategies and would be aware of the strategy they were assigned to. The outcome of interest would be incident endometrial cancer. Each woman would be followed from assignment (baseline) until the development of endometrial cancer, incomplete-follow-up, or 28 years after baseline, whichever occurs first. We defined incomplete follow-up as nonresponse to alcohol intake-related questions.

The causal contrasts of interest would be the intention-to-treat effect—that is, the effect of being assigned a strategy, regardless of whether women adhere to it—and the per-protocol effect—that is, the effect that would have been observed had all women adhered to their assigned strategy over the 28-year follow-up.

To estimate the intention-to-treat effect, we would conduct an intention-to-treat analysis that compares the 28-year risk (cumulative incidence) between the group assigned to each strategy. In the presence of incomplete follow-up, inverse probability weights (a function of baseline and time-varying prognostic factors) would be used to adjust for potential selection bias [11].

To estimate the per-protocol effect, one option is to conduct a per-protocol analysis that appropriately adjusts for baseline and time-varying prognostic factors that also predict adherence. In the absence of sufficient information on these factors, we could conduct a per-protocol analysis based on IV estimation, with the dichotomous randomization indicator as the proposed instrument. IV conditions (1) and (3) would be expected to hold by design, but we would need to assume that condition (2) holds. That is, we would need to assume that being aware of their treatment assignment did not affect participants’ behavior in ways that may affect the outcome. We would also need to assume a structural model on how different degrees of adherence—over time and in magnitude of alcohol intake—relate to the outcome. For example, we could use the SNCFTM described above to estimate the 28-risk risk of endometrial cancer in the study population under full adherence to each strategy.

Note that the per-protocol effect involves the dynamic strategy “always ½ drink per day, unless an absolute contraindication for moderate alcohol consumption arises” whereas our SNCFTM can only be used to compare static strategies such as “always ½ drink per day, regardless of contraindications for moderate alcohol consumption”. Therefore, our per-protocol analysis implicitly assumes that the incidence of contraindications is not high enough to substantially alter the per-protocol effect estimate.

### Target trial emulation

We emulated the above target trial using observational case-control data sampled from the NHS [14]. In brief, women aged 30–55 years from 11 U.S. states were enrolled in the NHS in 1976 upon completion of an initial questionnaire, and continuously followed up via biennial questionnaires on lifestyle and behavioral factors, as well as health outcomes. Our treatment, alcohol intake, was first assessed in 1980 using a validated semiquantitative food frequency questionnaire, and has been updated every 2 to 4 years. Alcohol intake values were truncated at the 99.5th percentile to eliminate implausible outliers. The outcome, incident endometrial cancer, was identified via biennial questionnaires or death records, and subsequently confirmed using medical records and pathology reports.

We applied the eligibility criteria of the target trial to women in the observational data. We additionally required women to have a measurement of alcohol intake at baseline (between the ages of 45 and 48) (Supplementary Fig. 4) and having contributed genotyping data used in any of 14 case-control studies of various disease outcomes, including endometrial cancer, that were nested in the NHS [16]. Follow-up started at the time of return of the first questionnaire after all eligibility criteria were met and ended as described above.

We constructed a weighted allele score of 23 single nucleotide polymorphisms (SNPs) that had a genome-wide significant association with alcohol intake and that did not have a genome-wide significant association for age of initiation of regular smoking, ever smoking, cigarettes per day, or smoking cessation (Supplementary Table 2) [17]. We then assumed that the value of this weighted score had been randomly assigned to eligible women.

To estimate an observational analogue of the intention-to-treat or per-protocol effects, we would proceed as for the target trial except that the randomization indicator would be replaced by a dichotomized version of the genetic score: women with low and high values of the genetic score would be assumed to have been assigned to strategy (1) and (2), respectively. An analogue of the intention-to-treat effect would be of little interest because of the low adherence to the assigned strategies in each level of the genetic score (26.8 and 2.9% among those assigned to strategy (1) and (2), respectively). This is the reason why most MR studies estimate an observational analogue of the per-protocol effect rather than an observational analogue of the intention-to-treat effect. To estimate the former, we used IV estimation as described for the target trial but with the continuous genetic score, rather than the dichotomous randomization indicator, as the proposed instrument. In our observational data, IV condition (1) holds (though weakly, see above), but we need to additionally assume conditions (2) and (3). Condition (2) requires that the genetic variants do not affect outcomes except via alcohol intake, which is trivially true for non-causal (surrogate) genetic variants. Condition (3) holds in the absence of shared causes, possibly arising from population stratification, of the genetic variants and endometrial cancer, and the genetic score be independent of the eligibility criteria (to prevent selection bias because the genotype is determined at conception but the eligibility criteria are defined decades later at the start of follow-up) [2, 18] and also to the matching factors in the case-control studies (which permits us to estimate E[*Z*| *Y*_0_ = 0] for the estimating equation). Among eligible women with genetic data, we used g-estimation of a structural nested cumulative failure time model with the blip function $\exp \left[{\gamma}_k\left({\overline{A}}_m;\psi \right)\right]=1+\frac{\exp \left(\psi {A}_m\right)-1}{k-m}$. The time-scale was in discrete 4-year age groups. Using the g-estimate $\hat{\psi}$, we estimated the marginal risk of endometrial cancer from age 45 to age 72 under the “never drink” strategy and the “always ½ drink per day” strategy among all eligible women in the NHS cohort. This study was approved by the Human Research Committees at Brigham and Women’s Hospital, Boston, MA, USA.

## Results

**Fig. 3:**
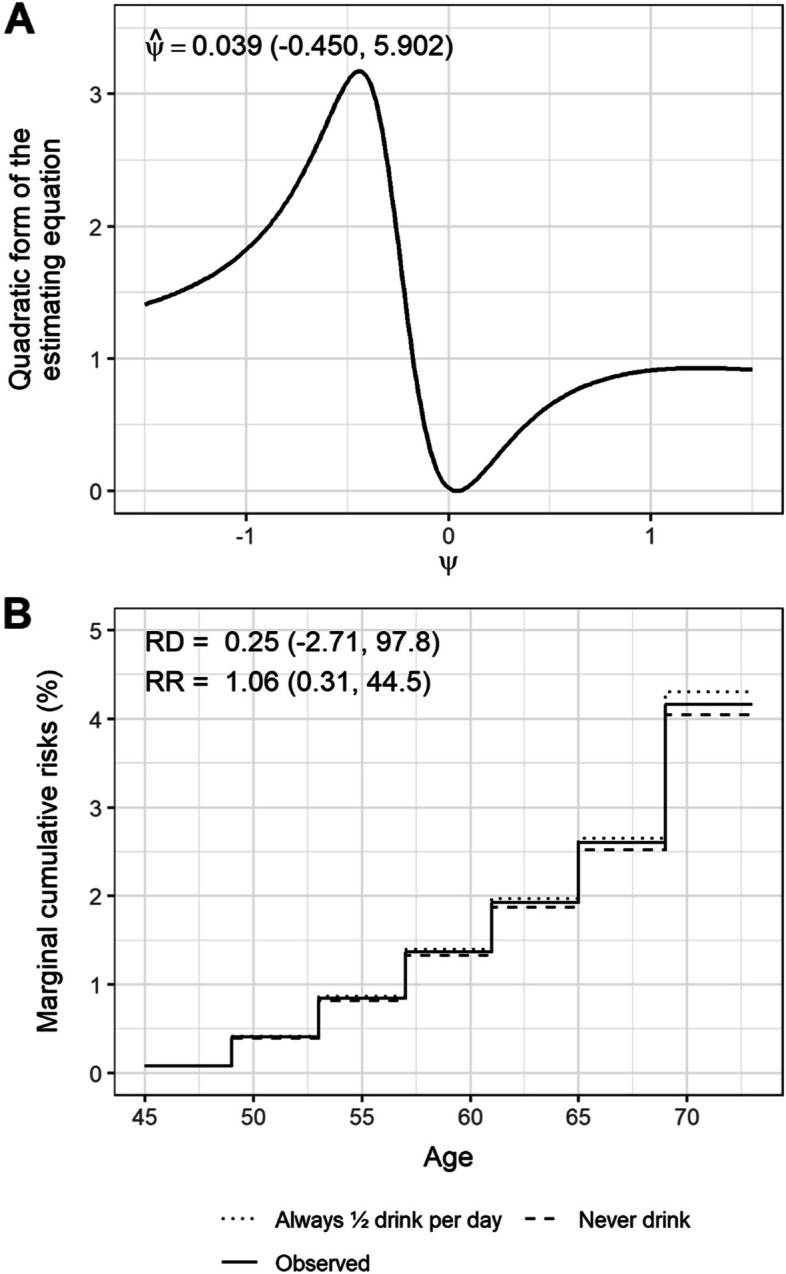
Plots of (**A**) the quadratic form of the estimating equation against possible *ψ* values and (**B**) the observed marginal cumulative risks and the marginal cumulative risks under the “never drink” and the “always ½ drink per day” strategies

Our analysis included 33,426 eligible women and genetic data was available for 6462 of them (Supplementary Table 1; Supplementary Fig. 4). Correlations between the weighted allele score and alcohol intake was about 0.06 across age groups (Supplementary Table 3A-B). Odds ratios for incident endometrial cancer per standard deviation increase in the weighted allele score ranged from 0.82 to 1.24 over follow-up (Supplementary Table 4). Our model converged in only 831 of 1000 bootstrap samples (no solution to the estimating equation could be found in the remaining bootstrap samples). The g-estimate $\hat{\psi}$ (95% confidence interval) was 0.039 (95% CI: − 0.450, 5.902), as shown by the point at which the quadratic form of the estimating equation reached a minimum (Fig. 3A). We used the g-estimate $\hat{\psi}$ to estimate the marginal risk of endometrial cancer from age 45 to age 72 in all eligible women, under the “never drink” strategy, and the “always ½ drink per day” strategy. We observed a risk difference of 0.3 percentage points (95% CI: − 2.7, 97.8) and a risk ratio of 1.06 (95% CI: 0.31, 44.5) (Fig. 3B).

## Discussion

Many observational studies which implement IV methods, including MR studies, involve an inherently time-varying treatment or exposure. Therefore, the goal of these studies is to estimate the effect of sustained treatment strategies. We described g-estimation of SNCFTMs for IV estimation of absolute risks under different treatment strategies, evaluated it in simulations, and implemented it as part of the observational emulation of a (hypothetical) target trial of alcohol intake interventions and endometrial cancer.

Our proposed method has several advantages: handling of continuous or dichotomous instruments and treatments, no restraints on the number of time points that can be included in the model, adjustment for selection bias due to loss to follow-up via inverse probability weighting, and application to case-control data without knowledge of sampling fractions. We discuss g-estimation of SNCFTMs using a time-fixed instrument, which is often the case in MR studies, but the method can be readily generalized to time-varying instruments (see Section S1 of the Supplementary material).

We demonstrated the validity of the method via simulations in simplified scenarios in which the effect was constant over time, did not vary across covariate levels, and in which only the most recent blip of treatment at time *k* had an effect on the outcome at time *k* + 1. Despite these simplifications, our simulations suffice to show a limitation of g-estimation of SNCFTMs: the SNCFTMs should only be used with rare outcomes because the expected conditional counterfactual risks must lie within the interval [0, 1], but the blip function leaves $\mathrm E\left[Y_k^{\left({\overline{\mathrm A}}_{\mathbf m-\mathbf1},\underline0\right)}\vert{\overline A}_m,Z,Y_m=0\right]$ unspecified and thus cannot impose bounds on it [10]. If the rare failure assumption does not hold, estimates may be invalid (see Remark 1 in reference [10]). Estimates for the marginal risks (4.2% in our observational data analysis) can be used to support the rare failure assumption. Under this assumption, it is irrelevant whether controls in the case-control studies are sampled using cumulative incidence sampling or incidence density sampling.

Our estimates of the effect of alcohol intake on the risk of endometrial cancer from age 45 to 75 had very wide 95% confidence intervals and our model did not converge in some of the bootstrap samples. This precludes us from making any substantive conclusions. The large variability of our estimate shows that informative MR analyses will ultimately require sample sizes much larger than ours—6492 women with genetic data, of whom only 219 developed endometrial cancer over follow-up—and/or stronger instruments. Previous analyses of the NHS data using Cox proportional hazards models reported an adjusted rate ratio of 0.88 (95% CI: 0.71, 1.09) when comparing moderate alcohol drinkers (5.0 to 14.9 g/day) to non-drinkers [19].

Especially given the width of the confidence intervals, we made three simplifications in our analysis that otherwise would have resulted in even more imprecise estimates. First, we assumed that IV condition [2] held in the presence of selection over the duration of follow-up and selection into the analytical sample. Second, we only considered one-dimensional parameter models. Multi-dimensional parameter models may include product terms between baseline covariates *L*_0_ and treatment (to allow for weaker versions of the homogeneity assumption) or product terms between time-varying covariates *L*_*m*_ and treatment (to compare dynamic strategies). The latter would require the development of blipping up procedures to obtain the risk under each dynamic strategy (previous descriptions of SNCFTMs have only described blipping up procedures for blip functions with time-fixed covariates) [10]. These procedures would require correct model specification for the nuisance functions $\mathrm E\left[Y_k^{\left({\overline A}_{m-1},\underline0\right)}\vert{\overline A}_m,{\overline L}_m,Z,Y_m=0\right]$ and the density of ${\overline{L}}_m$[10]. Third, we assumed marginal exchangeability for the instrument *Z* rather than conditional exchangeability, $Z\coprod Y_k^g\mid L_0$, where *L*_0_ is a vector of measured baseline covariates such that g-estimation is based on the conditional mean of the instrument, E[*Z*| *L*_0_, *Y*_0_ = 0].

## Conclusion

In summary, we have described how to conduct MR and other IV analyses with time-varying treatments and failure time outcomes using SNCFTMs. Our simulations confirm the validity of the proposed method and our data analysis indicate that these MR analyses require very large sample sizes. Larger databases are becoming increasingly available as genetic biobanks, such as the Million Veterans Program [20] and the UK Biobank [21], continue to collect detailed longitudinal data on non-genetic exposures and health outcomes. This work provides a basis for IV analyses of time-varying treatment and failure time outcomes in those databases.

## Supplementary Information

**Additional file 1: S1.** Structural nested cumulative failure time models with a time-varying instrumental variable. **Supplementary Figure 1.** Distributions of *ψ* estimates across 1,000 iterations using g-estimation with an instrumental variable under different data-generating mechanisms, different sample sizes (*n* = 10,000; *n* = 25,000 or *n* = 50,000), and different instrument-exposure strengths (*α*_*ZA*_ = 0.10; *α* = 0.25; or *α* = 0.45). The lower and upper hinges correspond to the 25th and 75th percentile. The lower and upper whiskers extend from the hinge to the smallest and largest values no further than 1.5*IQR from the hinge, where IQR is the interquartile range. The median is represented by the line between the hinges, and the mean is represented by the diamond point symbol. The percentages provided above each box plot represents the percentage of iterations in which the model did not converge. **Supplementary Figure 2.** A. Distributions of marginal risk differences across 1,000 iterations using different g-estimation approaches under different data-generating mechanisms with *λ* = 5%. The lower and upper hinges correspond to the 25th and 75th percentile. The lower and upper whiskers extend from the hinge to the smallest and largest values no further than 1.5*IQR from the hinge, where IQR is the interquartile range. The median is represented by the line between the hinges, and the mean is represented by the diamond point symbol. B. Distributions of marginal risk ratios across 1,000 iterations using different g-estimation approaches under different data-generating mechanisms with *λ* = 5%. The lower and upper hinges correspond to the 25th and 75th percentile. The lower and upper whiskers extend from the hinge to the smallest and largest values no further than 1.5*IQR from the hinge, where IQR is the interquartile range. The median is represented by the line between the hinges, and the mean is represented by the diamond point symbol. **Supplementary Figure 3.** A. Distributions of marginal risk differences across 1,000 iterations using different g-estimation approaches under different data-generating mechanisms with baseline hazards of 5%, 10% and 25%. The lower and upper hinges correspond to the 25th and 75th percentile. The lower and upper whiskers extend from the hinge to the smallest and largest values no further than 1.5*IQR from the hinge, where IQR is the interquartile range. The median is represented by the line between the hinges, and the mean is represented by the diamond point symbol. B. Distributions of marginal risk ratios across 1,000 iterations using different g-estimation approaches under different data-generating mechanisms with baseline hazards of 5%, 10% and 25%. The lower and upper hinges correspond to the 25th and 75th percentile. The lower and upper whiskers extend from the hinge to the smallest and largest values no further than 1.5*IQR from the hinge, where IQR is the interquartile range. The median is represented by the line between the hinges, and the mean is represented by the diamond point symbol. **Supplementary Figure 4.** Flowchart of inclusion and exclusion of Nurses’ Health Study participants. **Supplementary Table 1.** Characteristics of Nurses’ Health Study I participants at start of follow-up. **Supplementary Table 2.** SNPs identified as proposed instruments for alcohol intake. **Supplementary Table 3.** A. Associations of proposed instrument (weighted allele score) with alcohol intake across baseline five-year age groups. B. Distributions of alcohol intake and proportion of heavy drinkers across quartiles of the proposed instrument (weighted allele score) across baseline five-year age groups. **Supplementary Table 4.** Associations of the proposed instrument (weighted allele score) with hazard of endometrial cancer across baseline five-year age groups.

## Data Availability

Data that support the findings of this study are not publicly available to protect participants’ privacy and confidentiality. Further information including the procedures to obtain and access data from the Nurses’ Health Studies is described at https://www.nurseshealthstudy.org/researchers(contact email: nhsaccess@channing.harvard.edu). R code to implement structural nested cumulative failure time models for confounding adjustment or for instrumental variable analyses are available at https://github.com/joy-shi1/sncftms.

## Abbreviations

IV: Instrumental variable
MR: Mendelian randomization
NHS: Nurses’ Health Study
SNCFTM: Structural nested cumulative failure time model
SNP: Single nucleotide polymorphism
