# Supplementary material for: Instrumental variable estimation for a time-varying treatment and a time-to-event outcome via structural nested cumulative failure time models
Source: BMC Med Res Methodol. 2021 Nov 25;21:258. doi: 10.1186/s12874-021-01449-w (PMC8620657; doi:10.1186/s12874-021-01449-w)
Supplement: Supplementary file 1 — Additional file 1: S1. Structural nested cumulative failure time models with a time-varying instrumental variable. Supplementary Figure 1. Distributions of ψ estimates across 1,000 iterations using g-estimation with an instrumental variable under different data-generating mechanisms, different sample sizes (n = 10,000; n = 25,000 or n = 50,000), and different instrument-exposure strengths (αZA = 0.10; α = 0.25; or α = 0.45). The lower and upper hinges correspond to the 25th and 75th percentile. The lower and upper whiskers extend from the hinge to the smallest and largest values no further than 1.5*IQR from the hinge, where IQR is the interquartile range. The median is represented by the line between the hinges, and the mean is represented by the diamond point symbol. The percentages provided above each box plot represents the percentage of iterations in which the model did not converge. Supplementary Figure 2. A. Distributions of marginal risk differences across 1,000 iterations using different g-estimation approaches under different data-generating mechanisms with λ = 5%. The lower and upper hinges correspond to the 25th and 75th percentile. The lower and upper whiskers extend from the hinge to the smallest and largest values no further than 1.5*IQR from the hinge, where IQR is the interquartile range. The median is represented by the line between the hinges, and the mean is represented by the diamond point symbol. B. Distributions of marginal risk ratios across 1,000 iterations using different g-estimation approaches under different data-generating mechanisms with λ = 5%. The lower and upper hinges correspond to the 25th and 75th percentile. The lower and upper whiskers extend from the hinge to the smallest and largest values no further than 1.5*IQR from the hinge, where IQR is the interquartile range. The median is represented by the line between the hinges, and the mean is represented by the diamond point symbol. Supplementary Figure 3. A. Distributions of marg [file 12874_2021_1449_MOESM1_ESM.pdf]

Instrumental variable estimation for a time-varying treatment and a time-to-event outcome via structural nested cumulative failure time models: supplementary materials

Joy Shi, Sonja A. Swanson, Peter Kraft, Bernard Rosner, Immaculata De Vivo, Miguel A. Hernán

S1. Structural nested cumulative failure time models with a time-varying instrumental variable

S1.1 Identifying assumptions

Let Z_k represent the value of the instrument during the interval k for $k = 0,1,2, \ldots, K$. Let $\bar{Z}_k = (Z_0, Z_1, \ldots, Z_k)$ represent instrument history from time 0 to time k. The instrument Z_k must meet the three instrumental conditions: (1) the instrument is associated with the exposure, or $Z_k \perp\!\!\!\perp A_k$ does not hold for any k; (2) the instrument affects the outcome only through the treatment, or $Y_{i,k+1}^{\bar{z}_k,g} = Y_{i,k+1}^{\bar{z}'_k,g} = Y_{i,k+1}^{g}$ for all individuals i, k,$\bar{z}_k$, $\bar{z}'_k$, g; and (3) there are no common causes, or other sources of lack of exchangeability, between the instrument and the outcome, or $\bar{Z}_k \perp\!\!\!\perp \underline{Y}_{k+1}^{\bar{z}_k,g} | Y_k^{\bar{z}_{k-1},g} = 0$ for all z, k, g (1). The last two conditions, taken together, imply conditional exchangeability between the instrument and the counterfactual outcome under a given treatment regime, $\bar{Z}_k \perp\!\!\!\perp \underline{Y}_{k+1}^{g} | Y_k^{g} = 0$.

Some variation of a homogeneity assumption, such as no modification of the effect of treatment A_k on the outcome Y_{k+1} by the instrument Z_k on the multiplicative scale, is necessary for point estimation.

S1.2 G-estimation of structural nested cumulative failure time models with a time-varying instrumental variable

SNCFTMs compare the counterfactual risks at k under the strategies $(\bar{A}_m, \underline{0})$ and $(\bar{A}_{m-1}, \underline{0})$, for each time $m < k$, among individuals who are free of the outcome through m (i.e., $Y_m = 0$) and had treatment history $\bar{A}_m$ and the same covariate history through m. When using instrumental variable estimation with a time-varying IV, "covariate history" means instrument history:

$$\exp[\gamma_k(\bar{A}_m;\psi)] = \begin{cases} \dfrac{\mathrm{E}\left[Y_k^{(\bar{A}_m,\underline{0})}\middle|\bar{A}_m,\bar{Z}_m,Y_m = 0\right]}{\mathrm{E}\left[Y_k^{(\bar{A}_{m-1},\underline{0})}\middle|\bar{A}_m,\bar{Z}_m,Y_m = 0\right]} & \text{if } Y_m = 0 \\ 1 & \text{if } Y_m = 1 \end{cases}$$

The blip function, $\gamma_k(\bar{A}_m;\psi)$, remains unchanged from SNCFTMs with instrumental variable estimation with a time-fixed IV.

For IV estimation with a time-varying IV, the estimating function is (1,2):

$$U(\psi^{\dagger};Z) = \sum_{m=0}^{K}(1 - Y_m) \sum_{k=m+1}^{K+1} (Z_m - \mathrm{E}[Z_m|Y_m = 0])H_{m,k}(\psi^{\dagger})$$

where $H_{m,k}(\psi^{\dagger})$ is defined as

$$H_{m,k}(\psi^{\dagger}) = \begin{cases} Y_k \exp\left(- \sum_{j=m}^{k-1} \gamma_{j,k}\left(\bar{A}_j;\psi^{\dagger}\right)\right) & \text{if } Y_m = 0 \\ 1 & \text{if } Y_m = 1 \end{cases}$$

Under the above IV assumptions, the value $\hat{\psi}$ that solves $E[U(\psi^{\dagger};Z)] = 0$ is our g-estimate (1,2). To solve this equation, the Newton-Raphson procedure can be similarly applied. The procedure for computing marginal counterfactual risks under a given treatment strategy using the g-estimate $\hat{\psi}$ is unchanged.

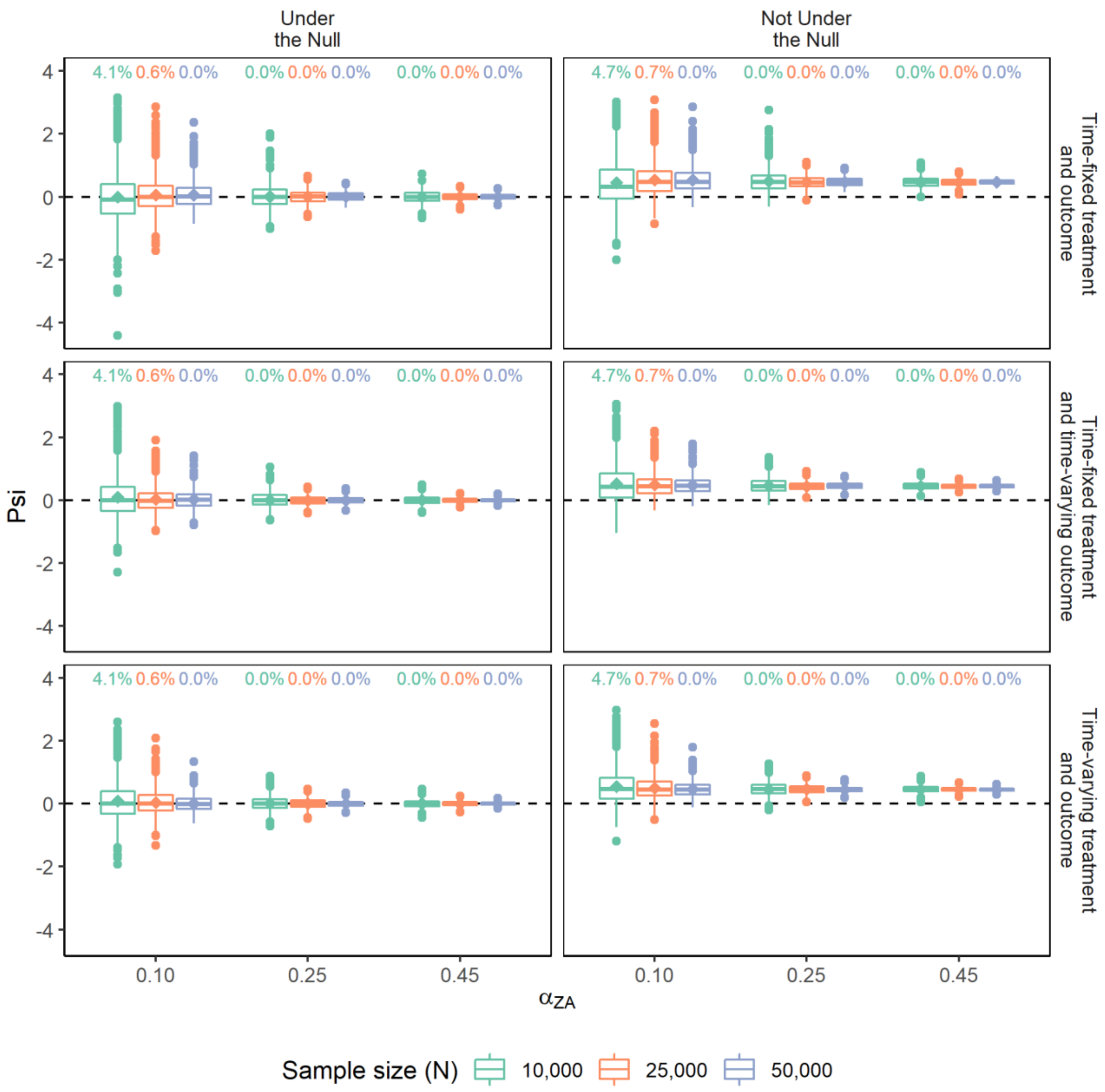

Supplementary Figure 1. Distributions of ψ estimates across 1,000 iterations using g-estimation with an instrumental variable under different data-generating mechanisms, different sample sizes (n = 10,000; n = 25,000 or n = 50,000), and different instrument-exposure strengths ($\alpha_{ZA} = 0.10$; $\alpha = 0.25$; or $\alpha = 0.45$). The lower and upper hinges correspond to the 25th and 75th percentile. The lower and upper whiskers extend from the hinge to the smallest and largest values no further than 1.5*IQR from the hinge, where IQR is the interquartile range. The median is represented by the line between the hinges, and the mean is represented by the diamond point symbol. The percentages provided above each box plot represents the percentage of iterations in which the model did not converge.

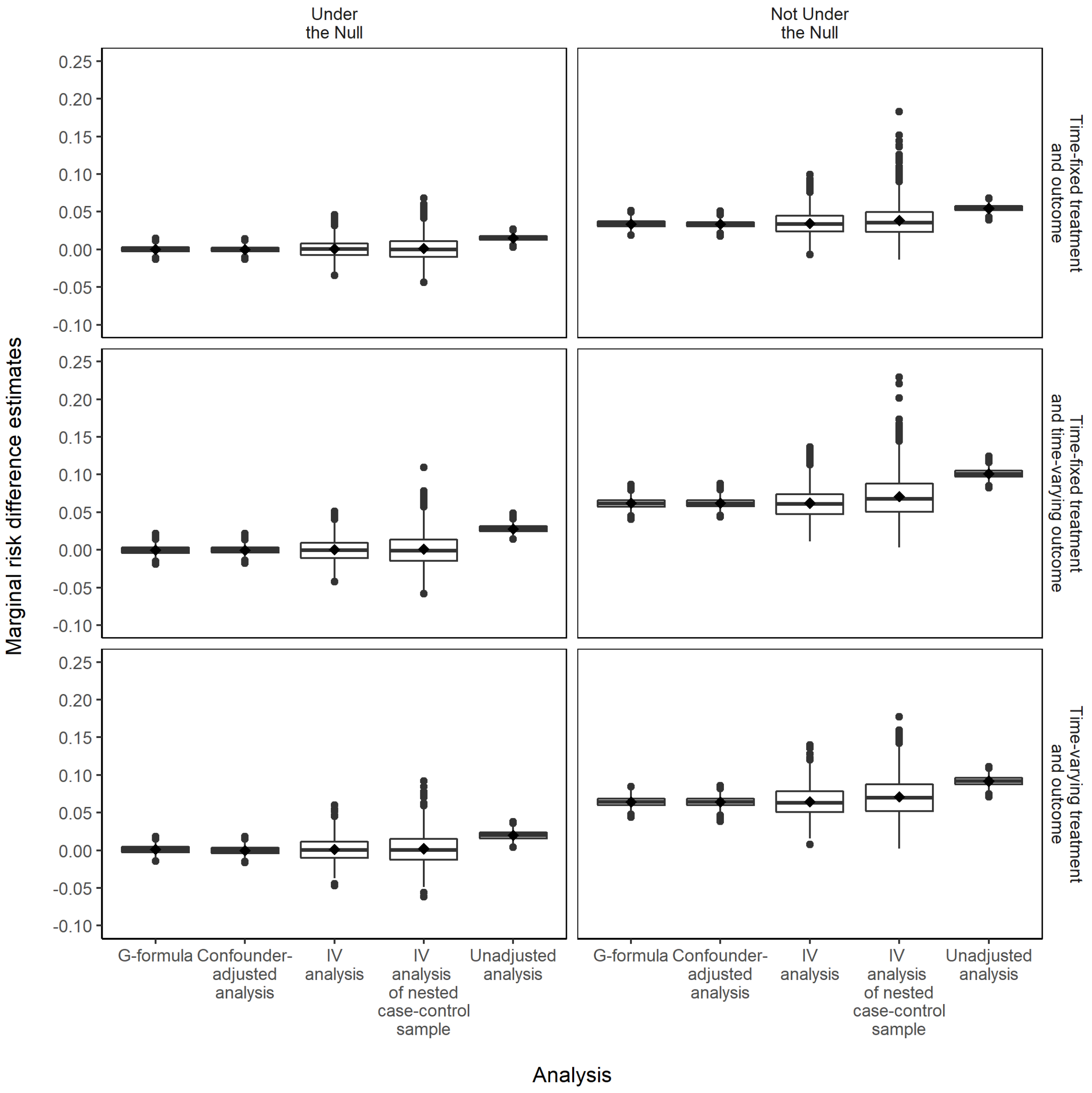

Supplementary Figure 2A. Distributions of marginal risk differences across 1,000 iterations using different g-estimation approaches under different data-generating mechanisms with $\lambda = 5\%$. The lower and upper hinges correspond to the 25th and 75th percentile. The lower and upper whiskers extend from the hinge to the smallest and largest values no further than 1.5*IQR from the hinge, where IQR is the interquartile range. The median is represented by the line between the hinges, and the mean is represented by the diamond point symbol.

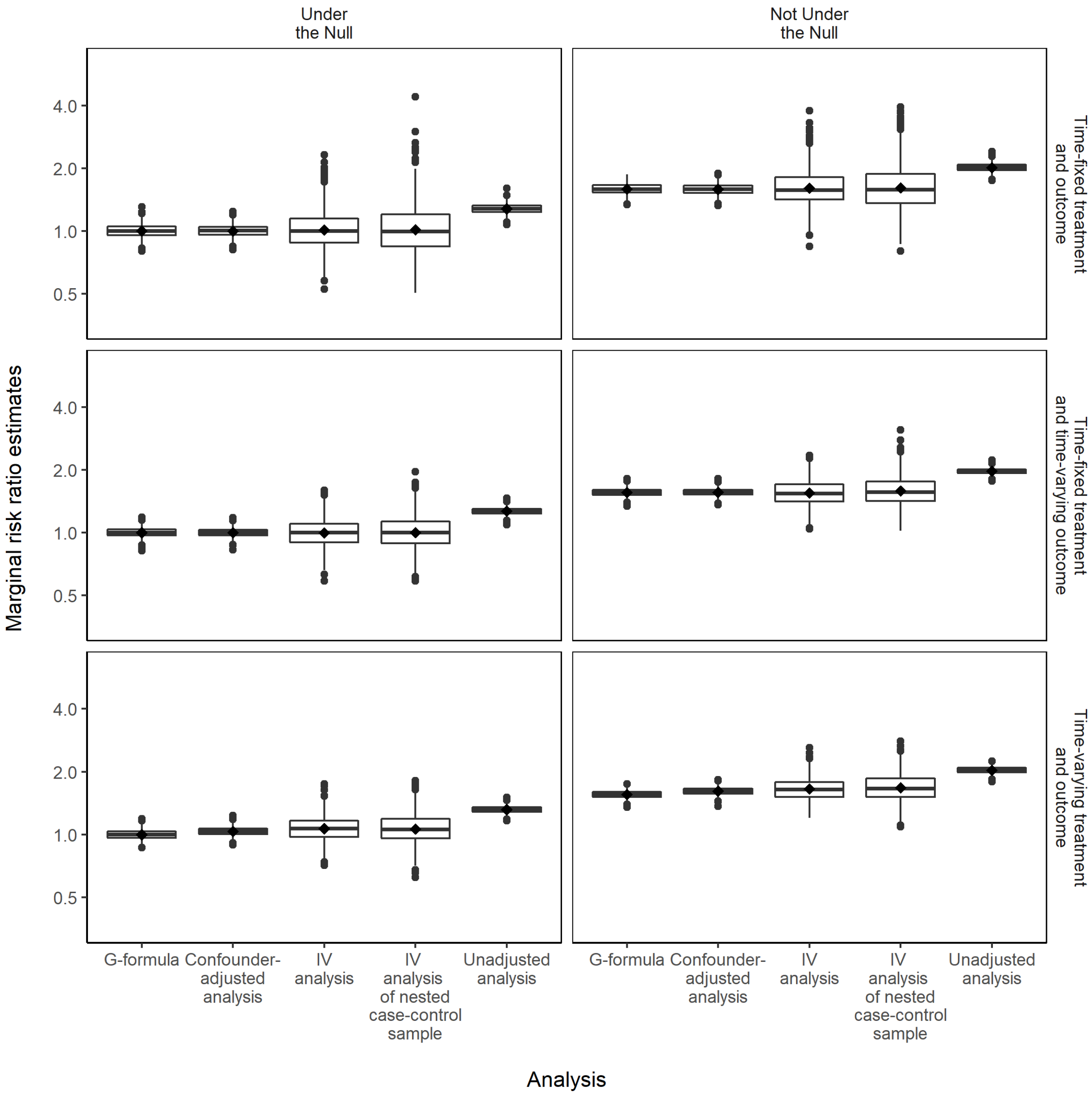

Supplementary Figure 2B. Distributions of marginal risk ratios across 1,000 iterations using different g-estimation approaches under different data-generating mechanisms with $\lambda = 5\%$. The lower and upper hinges correspond to the 25th and 75th percentile. The lower and upper whiskers extend from the hinge to the smallest and largest values no further than 1.5*IQR from the hinge, where IQR is the interquartile range. The median is represented by the line between the hinges, and the mean is represented by the diamond point symbol.

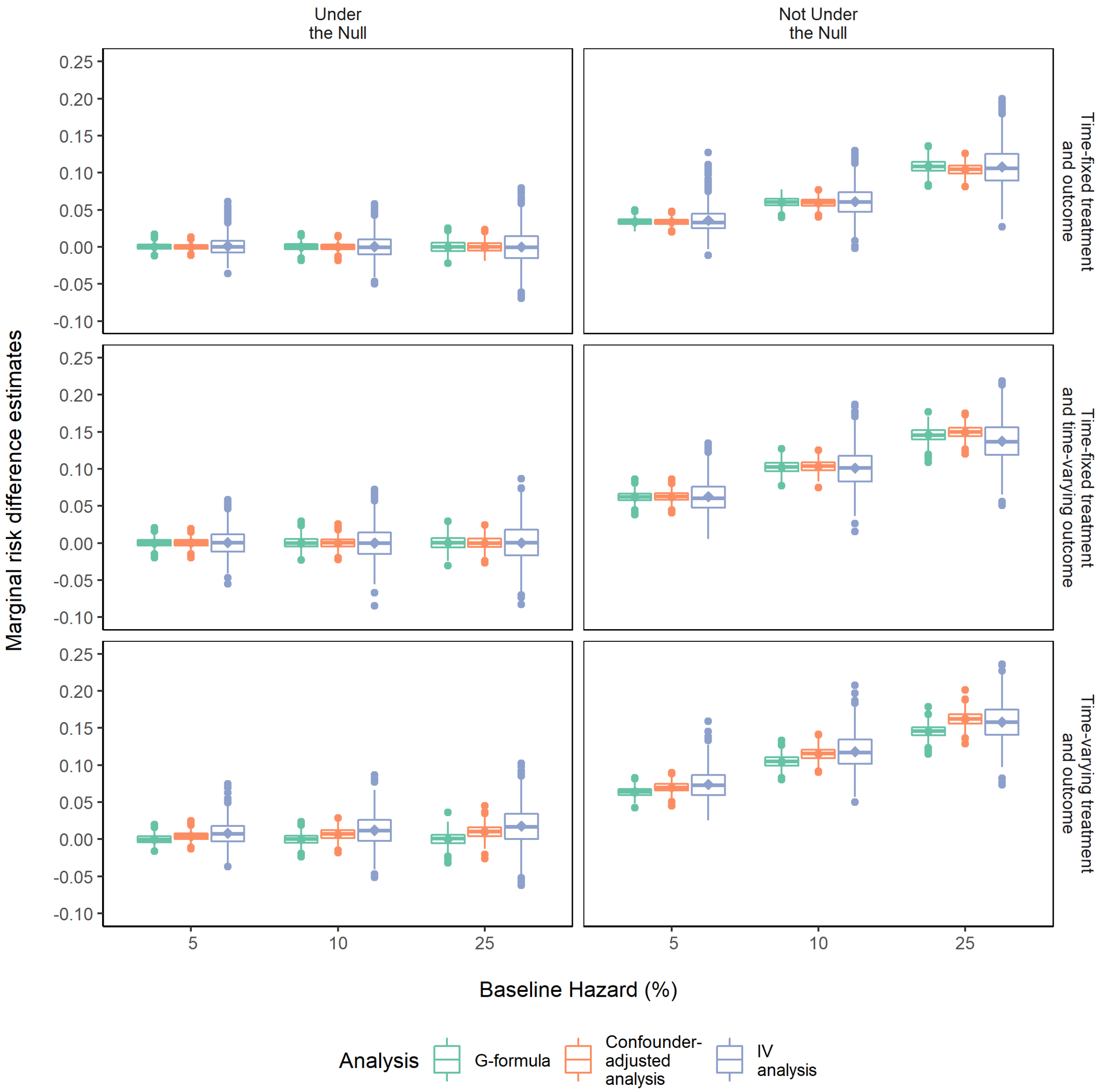

Supplementary Figure 3A. Distributions of marginal risk differences across 1,000 iterations using different g-estimation approaches under different data-generating mechanisms with baseline hazards of 5%, 10% and 25%. The lower and upper hinges correspond to the 25th and 75th percentile. The lower and upper whiskers extend from the hinge to the smallest and largest values no further than 1.5*IQR from the hinge, where IQR is the interquartile range. The median is represented by the line between the hinges, and the mean is represented by the diamond point symbol.

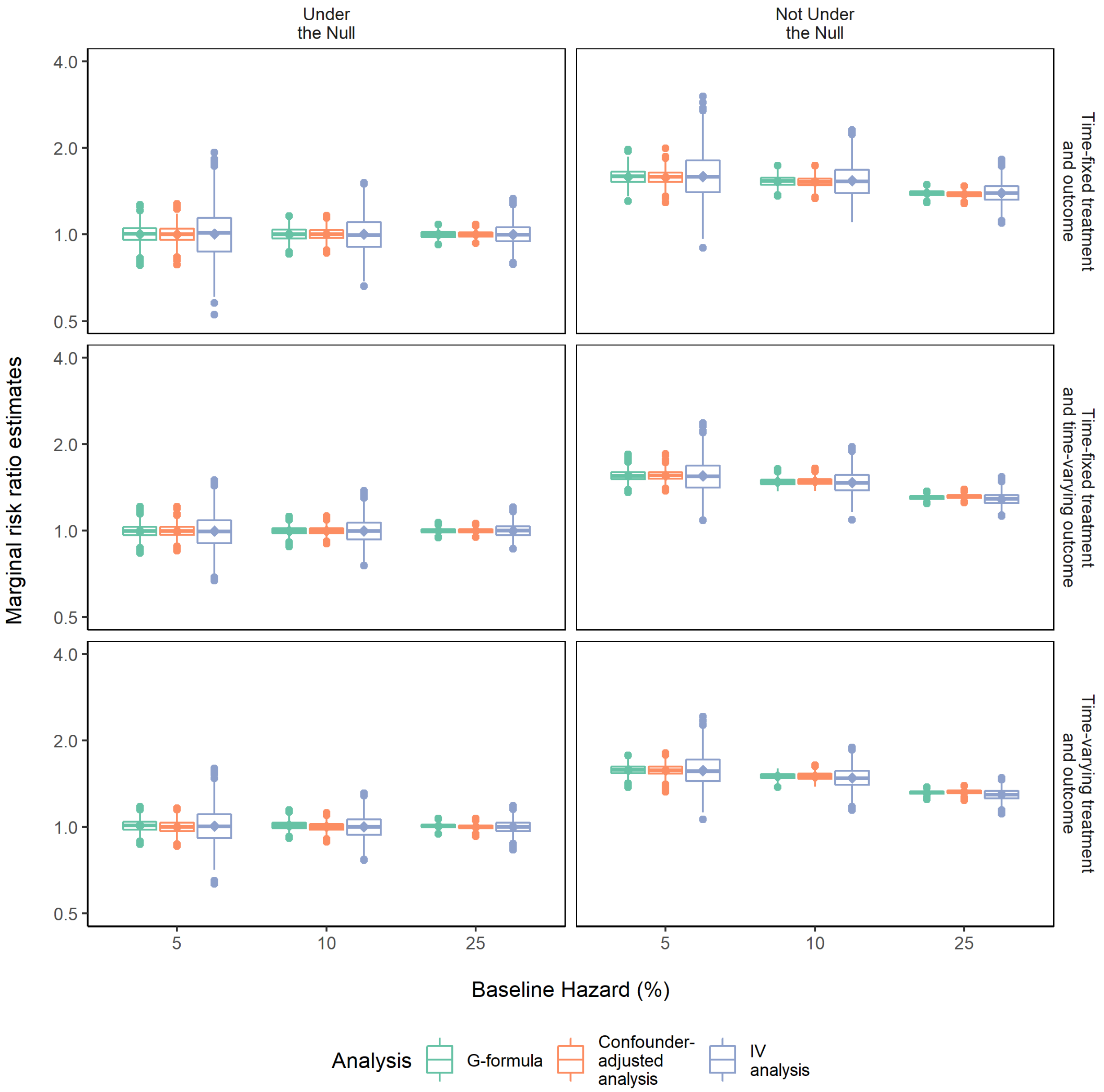

Supplementary Figure 3B. Distributions of marginal risk ratios across 1,000 iterations using different g-estimation approaches under different data-generating mechanisms with baseline hazards of 5%, 10% and 25%. The lower and upper hinges correspond to the 25th and 75th percentile. The lower and upper whiskers extend from the hinge to the smallest and largest values no further than 1.5*IQR from the hinge, where IQR is the interquartile range. The median is represented by the line between the hinges, and the mean is represented by the diamond point symbol.

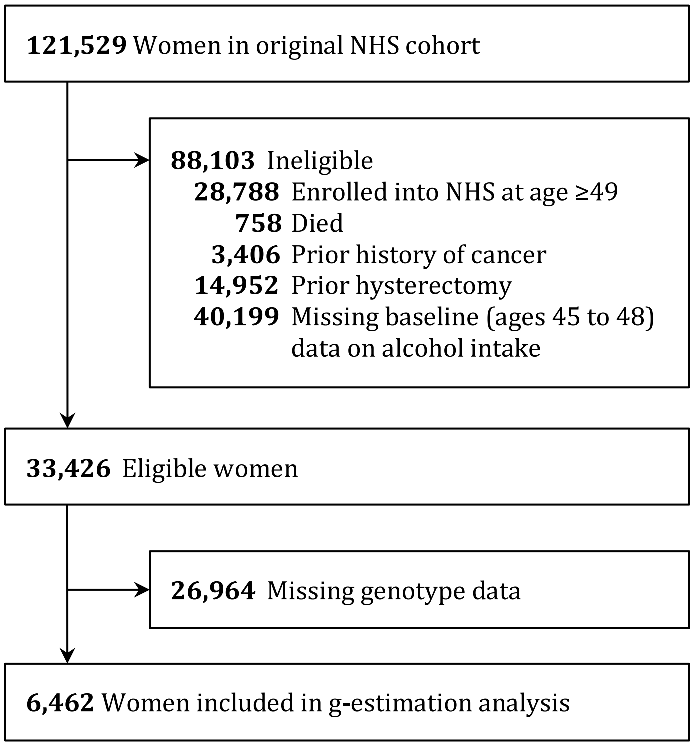

Supplementary Figure 4. Flowchart of inclusion and exclusion of Nurses' Health Study participants

Supplementary Table 1. Characteristics of Nurses' Health Study I participants at start of follow-up

Characteristic, summary measure	Full cohort (n = 33,426)		Genetic cohort (n = 6,462)	
	N (% missing)	Summary statistic	N (% missing)	Summary statistic
Age (years), mean ± SD	33,426 (0.0)	46.5 ± 1.14	6,462 (0.0)	46.5 ± 1.14
BMI, n (%)	32,733 (2.1)		6,355 (1.7)	
< 25 kg/m^2		20,182 (61.7)		3,942 (62.0)
25 to <30 kg/m^2		8,173 (25.0)		1,571 (24.7)
≥ 30 kg/m^2		4,378 (13.4)		842 (13.2)
Hispanic ethnicity, n (%)	33,426 (0.0)	245 (0.7)	6,462 (0.0)	24 (0.4)
Menarche to menopause (years), mean ± SD	32,478 (2.8)	33.8 ± 2.33	6,383 (1.2)	33.9 ± 2.22
Postmenopausal, n (%)	32,478 (2.8)	4,731 (14.6)	6,383 (1.2)	840 (13.2)
Years since menopause among postmenopausal women, median (IQR)	4,731 (0.0)	1 (0, 3)	840 (0.0)	1 (0, 3)
Ever hormone therapy use, n (%)				
Estrogen-only	33,426 (0.0)	354 (1.1)	6,462 (0.0)	63 (1.0)
Estrogen + progesterone	33,426 (0.0)	494 (1.5)	6,462 (0.0)	78 (1.2)
Other	33,422 (0.0)	319 (1.0)	6,461 (0.0)	51 (0.8)
Ever oral contraceptive use, n (%)	33,202 (0.7)	20,808 (62.7)	6,440 (0.3)	4,061 (63.1)
Years of oral contraceptive use among ever users, median (IQR)	20,777 (0.1)	35 (12, 77)	4,058 (0.1)	35 (12, 76)
Nulliparous, n (%)	32,928 (1.5)	0 (0.0)	6,404 (0.9)	0 (0.0)
Parity, median (IQR)	32,928 (1.5)	3 (2, 4)	6,404 (0.9)	3 (2, 4)
Smoking status, n (%)	33,364 (0.2)		6,449 (0.2)	
Never smoker		14,527 (43.5)		2,863 (44.4)
Former smoker		10,716 (32.1)		2,242 (34.8)
Current smoker		8,121 (24.3)		1,344 (20.8)
Pack-years smoking, median (IQR)	33,020 (1.2)	2 (0, 18)	6,370 (1.4)	2 (0, 16)
Diabetic, n (%)	33,426 (0.0)	684 (2.0)	6,462 (0.0)	162 (2.5)
Family history of endometrial cancer, n (%)	28,217 (15.6)	1,622 (5.7)	5,948 (8.0)	338 (5.7)
Alcohol intake (g/day), median (IQR)	33,426 (0.0)	1.8 (0, 7.8)	6,462 (0.0)	2 (0, 9)

Supplementary Table 2. SNPs identified as proposed instruments for alcohol intake

Chr	Position	rsID	Ref. allele	Alt. allele	Alt. allele frequency	Weight[a]	Affymetrix		HumanCore Exome		Illumina HapMap		OmniExpress		OncoArray		Excluded
							Imputed or genotyped	R^2	Imputed or genotyped	R^2	Imputed or genotyped	R^2	Imputed or genotyped	R^2	Imputed or genotyped	R^2	
1	4548453	rs705687	A	G	0.796	-0.0109	Imputed	1.00	Imputed	0.99	Imputed	0.99	Imputed	0.99	Imputed	0.99	
1	165119792	rs10753661	G	A	0.679	-0.0086	Imputed	1.00	Imputed	1.00	Imputed	1.00	Imputed	0.99	Imputed	1.00	
2	144225215	rs13024996	C	A	0.364	-0.0109	Imputed	0.99	Imputed	0.99	Imputed	0.99	Imputed	0.99	Imputed	1.00	
3	141124607	rs2011092	T	C	0.334	-0.0089	Imputed	1.00	Imputed	0.99	Imputed	0.99	Imputed	1.00	Genotyped	1.00	
3	141267295	rs60654199	C	A	0.064	-0.0167	Imputed	0.97	Imputed	0.96	Imputed	0.98	Imputed	0.98	Imputed	0.98	
4	39406254	rs7682824	C	T	0.205	0.0084	Imputed	0.59	Imputed	0.60	Imputed	0.61	Imputed	0.64	Imputed	0.61	Y [b]
4	39414993	rs11940694	A	G	0.596	0.0259	Genotyped	1.00	Genotyped	1.00	Imputed	0.98	Genotyped	1.00	Genotyped	1.00	
4	39418965	rs35538052	G	A	0.384	-0.0085	Imputed	0.97	Imputed	0.99	Imputed	1.00	Imputed	1.00	Imputed	1.00	Y [c]
4	42151306	rs4501255	C	G	0.233	0.0107	Imputed	0.98	Imputed	0.98	Imputed	1.00	Imputed	0.99	Imputed	0.98	
4	171086393	rs12651313	C	G	0.433	-0.0086	Imputed	1.00	Imputed	1.00	Imputed	1.00	Imputed	1.00	Imputed	1.00	
5	144412335	rs12655091	G	A	0.523	-0.0083	Imputed	1.00	Imputed	1.00	Imputed	1.00	Genotyped	1.00	Imputed	1.00	
7	73042443	rs6460047	T	C	0.211	0.0116	Imputed	0.95	Imputed	0.97	Imputed	0.95	Imputed	0.99	Imputed	0.97	
7	103840115	rs35034355	G	A	0.516	-0.0081	Imputed	1.00	Imputed	1.00	Imputed	1.00	Imputed	1.00	Imputed	1.00	
8	20949917	rs13250583	C	T	0.206	-0.0097	Imputed	1.00	Imputed	0.98	Imputed	0.99	Imputed	0.99	Imputed	0.98	
10	110507806	rs7074871	G	A	0.255	-0.0094	Imputed	1.00	Imputed	1.00	Imputed	1.00	Imputed	1.00	Imputed	1.00	
11	8642218	rs7950166	C	T	0.630	-0.0098	Imputed	1.00	Imputed	1.00	Imputed	0.98	Imputed	1.00	Imputed	0.97	
12	81601464	rs10506274	G	T	0.484	-0.0090	Imputed	1.00	Imputed	1.00	Imputed	1.00	Imputed	0.99	Imputed	1.00	
12	92170791	rs4842786	G	A	0.580	-0.0088	Imputed	1.00	Imputed	0.92	Imputed	0.98	Genotyped	1.00	Genotyped	1.00	
13	27124360	rs500321	A	T	0.739	-0.0097	Imputed	1.00	Imputed	0.99	Imputed	1.00	Imputed	1.00	Imputed	0.99	
14	94844947	rs28929474	C	T	0.017	-0.0368	Imputed	0.97	Genotyped	1.00	Imputed	0.98	Imputed	0.97	Imputed	0.96	
15	86796012	rs12907323	A	G	0.422	0.0085	Imputed	1.00	Imputed	0.99	Imputed	0.99	Imputed	0.99	Imputed	0.99	
16	20013793	rs2764771	G	A	0.299	0.0099	Genotyped	1.00	Imputed	0.97	Imputed	0.97	Imputed	0.97	Imputed	0.97	
16	28754684	rs378421	G	A	0.406	-0.0112	Imputed	0.96	Imputed	0.96	Imputed	0.96	Imputed	0.96	Imputed	0.96	
16	73912588	rs1104608	G	C	0.419	-0.0110	Imputed	0.96	Imputed	0.76	Imputed	0.93	Imputed	0.96	Imputed	0.78	Y [b]
18	55080437	rs4092465	A	G	0.634	-0.0083	Imputed	0.96	Imputed	0.68	Genotyped	1.00	Genotyped	1.00	Imputed	0.96	Y [b]
19	49214274	rs281379	G	A	0.498	0.0137	Imputed	0.96	Genotyped	1.00	Imputed	0.98	Imputed	0.98	Imputed	0.98	

[a] Weights are from betas reported in Liu et al. (2019)

[b] Excluded due to low (<0.90) imputation R^2

[c] Excluded due to LD-based clumping

Supplementary Table 3A. Associations of proposed instrument (weighted allele score) with alcohol intake across baseline five-year age groups

Age	n	Alcohol intake (g/day)	
		r (95% CI)	β (95% CI) [a]
45-48	6,462	0.059 (0.035, 0.083)	0.623 (0.366, 0.880)
49-52	6,037	0.055 (0.030, 0.080)	0.569 (0.308, 0.831)
53-56	5,688	0.060 (0.034, 0.086)	0.579 (0.327, 0.831)
57-60	5,441	0.068 (0.041, 0.094)	0.687 (0.417, 0.956)
61-64	5,137	0.061 (0.034, 0.089)	0.664 (0.369, 0.959)
65-68	4,726	0.073 (0.045, 0.101)	0.864 (0.528, 1.201)
69-72	3,507	0.058 (0.025, 0.091)	0.695 (0.302, 1.088)

[a] Change in alcohol intake (g/day) per standard deviation increase in the weighted allele score

Supplementary Table 3B. Distributions of alcohol intake and proportion of heavy drinkers across quartiles of the proposed instrument (weighted allele score) across baseline five-year age groups

Age	Quartile 1		Quartile 2		Quartile 3		Quartile 4	
	n	Alcohol intake (g/day), mean ± SD	n	Alcohol intake (g/day), mean ± SD	n	Alcohol intake (g/day), mean ± SD	n	Alcohol intake (g/day), mean ± SD
45-48	1,616	5.77 ± 9.52	1,615	6.65 ± 10.63	1,615	6.48 ± 10.59	1,616	7.40 ± 11.36
49-52	1,501	5.55 ± 9.73	1,518	6.35 ± 10.47	1,504	6.28 ± 10.33	1,514	7.15 ± 10.87
53-56	1,412	4.93 ± 8.76	1,438	5.66 ± 9.69	1,417	6.15 ± 9.95	1,421	6.49 ± 10.25
57-60	1,352	5.14 ± 8.81	1,384	5.93 ± 10.13	1,351	6.01 ± 10.02	1,354	6.97 ± 11.36
61-64	1,271	5.66 ± 9.41	1,325	6.54 ± 10.95	1,269	6.61 ± 10.81	1,272	7.38 ± 11.64
65-68	1,173	6.14 ± 10.20	1,208	7.12 ± 11.70	1,165	7.22 ± 11.57	1,180	8.57 ± 13.27
69-72	883	6.12 ± 11.14	894	7.21 ± 11.68	872	7.20 ± 11.69	858	8.01 ± 12.66

Supplementary Table 4. Associations of the proposed instrument (weighted allele score) with hazard of endometrial cancer across baseline five-year age groups

Age	n	Number of events	OR (95% CI) [a]
45-48	6,462	13	0.815 (0.474, 1.403)
49-52	6,037	30	0.990 (0.692, 1.418)
53-56	5,688	25	1.048 (0.707, 1.553)
57-60	5,441	48	1.244 (0.934, 1.657)
61-64	5,137	44	0.852 (0.632, 1.147)
65-68	4,726	33	0.997 (0.707, 1.407)
69-72	3,507	32	0.891 (0.628, 1.263)

[a] Odds ratio for incident endometrial cancer per standard deviation increase in the weighted allele score

References

1. Hernán MA, Robins JM. Causal Inference: What If. Boca Raton: Chapman & Hall/CRC; 2020.

2. Picciotto S, Hernán MA, Page JH, Young JG, Robins JM. Structural nested cumulative failure time models to estimate the effects of interventions. J Am Stat Assoc. 2012;107(499):886–900.
